# Silver Doped Mesoporous Silica Nanoparticles Based Electrochemical Enzyme-Less Sensor for Determination of H_2_O_2_ Released from Live Cells

**DOI:** 10.3390/mi10040268

**Published:** 2019-04-21

**Authors:** Danting Yang, Ning Ni, Lu Cao, Xin Song, Yasmin Alhamoud, Guangxia Yu, Jinshun Zhao, Haibo Zhou

**Affiliations:** 1 Department of Preventative Medicine, Zhejiang Provincial Key Laboratory of Pathological and Physiological Technology, Medical School of Ningbo University, Ningbo 315211, China; nn15058476109@gmail.com (N.N.); 1611101232@nbu.edu.cn (X.S.); yasmeenfood@hotmail.com (Y.A.); yuguangxia@nbu.edu.cn (G.Y.); zhaojinshun@nbu.edu.cn (J.Z.); 2 Institute of Pharmaceutical Analysis and Guangdong Province Key Laboratory of Pharmacodynamic Constituents of Traditional Chinese Medicine & New Drug Research, College of Pharmacy, Jinan University, Guangzhou 510632, China; 3 School of Life Sciences, Sun Yat-sen University, Guangzhou 510275, China; caolu5@mail2.sysu.edu.cn

**Keywords:** silver-mesoporous silica nanoparticles, hydrogen peroxide, electrochemical sensor, live cells

## Abstract

In this study, a silver doped mesoporous silica nanoparticles-based enzyme-less electrochemical sensor for the determination of hydrogen peroxide (H_2_O_2_) released from live cells was constructed for the first time. The presented electrochemical sensor exhibited fast response (2 s) towards the reduction of H_2_O_2_ concentration variation at an optimized potential of −0.5 V with high selectivity over biological interferents such as uric acid, ascorbic acid, and glucose. In addition, a wide linear range (4 μM to 10 mM) with a low detection limit (LOD) of 3 μM was obtained. Furthermore, the Ag-mSiO_2_ nanoparticles/glass carbon electrode (Ag-mSiO_2_ NPs/GCE) based enzyme-less sensor showed good electrocatalytic performance, as well as good reproducibility, and long-term stability, which provided a successful way to in situ determine H_2_O_2_ released from live cells. It may also be promising to monitor the effect of reactive oxygen species (ROS) production in bacteria against oxidants and antibiotics.

## 1. Introduction

The rapid, specific, and accurate hydrogen peroxide (H_2_O_2_) determination is quite essential in bioanalytical fields. H_2_O_2_, as the most common representative of reactive oxygen species, which endogenously produced in a cell, is a key player in various pathological processes. High levels of H_2_O_2_ results in not only tissue and DNA damage, but also aging, diabetes, cancer, traumatic brain injury, and neurodegenerative disorders [1,2]. Consequently, the determination of H_2_O_2_ attracted much interest as its potential as an indicator of oxidative stress-related diseases. Several analytical methods such as spectrophotometry [3], fluorimetry [4], titrimetry [5], chromatography [6], and electrochemistry [7,8] have been applied for highly sensitive H_2_O_2_ determination. Amongst the above techniques, electrochemical methods are always optimal choices, due to their high sensitivity, operational simplicity, fast response, low cost, portability, and multi-analyte detection [9,10]. However, most electrode modifiers possessed high potentials, which limited the sensitive and selective performance of H_2_O_2_ at ordinary solid electrodes [11]. Thus, the electrode modification is of great importance to minimize the overpotential of redox reaction and improve the rate of electron transfer.

Reportedly, noble metal nanoparticles [12], noble metal nanoparticles on carbon nanostructures [13], and bimetallic nanoparticles [14] have been used for the electrode surfaces modification because of their unique catalytic and electronic properties. The constructed electrochemical sensors performed a highly electrocatalytic activity toward H_2_O_2_ reduction or oxidization. Compared with the enzyme-based sensor, enzyme-less sensors are specific to analyte and overcome the limitations from enzyme, such as high cost, susceptible conformation affected by pH, temperature, etc. [15]. Silver nanoparticles (Ag NPs) exhibit good synergetic effect on H_2_O_2_ reduction [16,17], which is widely used for enzyme-less electrochemical sensors of H_2_O_2_. However, Ag NPs are easy to aggregate in solution before making of electrodes, reducing the sensitivity of the electrochemical sensor. Use of mesoporous silica nanoparticles (mSiO_2_ NPs) is one of the most effective ways to prevent aggregation [18] because Ag NPs can embed uniformly in the pores of mSiO_2_ NPs. In addition, the mSiO_2_ NPs possess unique properties such as large surface area, uniform pore size, ordered mesostructure, and facile surface modification [19,20], which make it a suitable electrically conductive host of Ag NPs to construct electrochemical sensors [21,22]. For example, Khan and Bandyopadhyaya [23] presented an enzyme-less amperometric H_2_O_2_ sensor based on Ag NPs impregnated amine functionalized mSiO_2_ NPs with a wider linearity range (5.3–124.3 mM). Azizi et al. [24] reported a new Ag-doped SBA-16 NPs modified carbon paste electrode, which could achieve detection of H_2_O_2_ in two linear ranges (20 μM to 8 mM, and 8 to 20 mM). The biosensor constructed by Ensafi et al. [10] could achieve a lower LOD of 0.45 μM. While the significant success has been made in the detection of traces of H_2_O_2_, which is summarized in the new work by Viter and Iatsunskyi [25], biosensors based on Ag-doped MCM-41 mSiO_2_ NPs for real-time detection of H_2_O_2_ released from live cells has not been reported yet, to the best of our knowledge.

**Figure 1:**
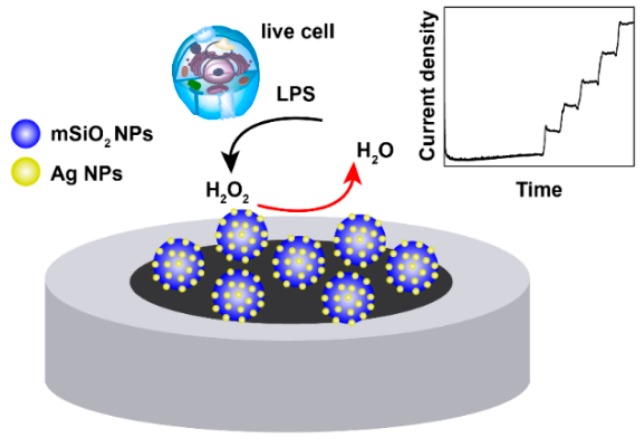
Schematic of electrode modified with silver-mesoporous silica nanoparticles (Ag-mSiO_2_ NPs) for H_2_O_2_ detection.

We presented an enzyme-less electrochemical biosensor based on Ag-doped MCM-41 mSiO_2_ NPs to determine H_2_O_2_ released from live cells (Figure 1). Our result showed a high electrocatalytic performance of Ag-mSiO_2_ nanoparticles/glass carbon electrode (NPs/GCE) towards H_2_O_2_ reduction. Additionally, a good linear range from 4 μM to 10 mM with a LOD of 3 μM was obtained. The presented sensor showed high selectivity over biological interferents, such as ascorbic acid, uric acid, and glucose. Moreover, H_2_O_2_ released from pheochromocytoma (PC-12) cells could be successfully detected using the present sensor. In addition, as reactive oxygen species (ROS) including H_2_O_2_ production in bacteria such as *Escherichia coli* and *Vaginal Lactobacilli* can increase the bacteria’s susceptibility to oxidative attack from antibiotic treatment [26,27], our sensor is promising to help understanding the mechanism of H_2_O_2_ producing by bacteria against antibiotics in a clinic. 

## 2. Materials and Methods 

### 2.1. Materials

Tetraethylorthosilicate (TEOS), hexadecyltrimethylammonium bromide (CTAB), formalin (HCHO, 37%), and silver nitrate (AgNO_3_) were purchased from Sigma Aldrich. N-(aminoethyl)-amino-propyl trimethoxysilane (TSD) was bought from Aladdin Company. Methanol (CH_3_OH), disodium hydrogen phosphate (Na_2_HPO_4_·12H_2_O), sodium dihydrogen phosphate (NaH_2_PO_4_·2H_2_O), sodium hydroxide (NaOH), potassium chloride (KCl), potassium ferricyanide (K_3_Fe(CN)_6_), ammonium nitrate (NH_4_NO_3_), and ethanol (C_2_H_5_OH) were purchased from Beijing Chemical Plant. All chemicals were analytical grade. Distilled water (18.2 MΩ cm^−1^) was used as a solvent. 

### 2.2. Preparation of Ag-mSiO_2_ Nanoparticles (NPs)

Ag-mSiO_2_ NPs was prepared following slight modification of the Tian reaction [28]. A quantity of 0.5 mL 2M NaOH was added in 55 mL 7.5 mM CTAB aqueous solution to make solution A; 1 mL TEOS was added to 5 mL methanol to make solution B; 0.25 mL of TSD was added to 5 mL 35 mM AgNO_3_ solution to make solution C. Under vigorous stirring, solution A was heated to 75 °C and added with 4 mL of solution B dropwise to react for 15 min. Then, solution C and 2 mL of solution B were added dropwise and reacted for 2 h. A total of 2.5 mL formalin solution was finally added and reacted for 1 h. The resulted NPs were removed from CTAB in NH_4_NO_3_ ethanol solution (50 mL) at 60 °C for 10 h under stirring. The resulted Ag-mSiO_2_ NPs were centrifuged, washed with distilled water thrice, and re-suspended into 50 mL deionized water for further use. 

### 2.3. Preparation of H_2_O_2_ Biosensors

The surface of a glassy carbon electrode (GCE, 3 mm in diameter) was firstly polished and cleaned to a mirror-like status. Different volumes of Ag-mSiO_2_ NPs suspension (10, 8, 5, and 3 μL) were dropped respectively on the pretreated electrode surface and dried in the air for 1 h. Then GCE modified with a stable Ag-mSiO_2_ NPs film (Ag-mSiO_2_ NPs/GCE) was used for the following electrochemical experiments.

### 2.4. Determination of H_2_O_2_

#### 2.4.1. Detection of Standard H_2_O_2_ Solution

H_2_O_2_ standard solutions with different concentrations were added to 10 mL of 0.2 M phosphate buffered saline (PBS, pH 6.8, saturated with N_2_) under stirring, for H_2_O_2_ detection. The applied potential (−0.40, −0.45, −0.50, and −0.55 V) was optimized for amperometric analysis of H_2_O_2_. The background current was firstly recorded in PBS (pH 6.8) without H_2_O_2_ and subtracted for H_2_O_2_ calibration plot construction. 

#### 2.4.2. Detection of H_2_O_2_ Released from Pheochromocytoma cells 

The pheochromocytoma (PC-12) cells were grown in 75 cm^2^ flasks containing RPMI-1640 medium including fetal bovine serum (10%), as well as penicillin and streptomycin (100 μg·mL^−1^) in 5% CO_2_ atmosphere at 37 °C. When the PC-12 cells reached a 90% confluence growth, they were centrifuged and responded into 4 mL of PBS (0.2 M pH 6.8). The cell-packed pellet of 1.0–2.0 × 10^5^ cells·cm^−2^ was obtained for amperometric analysis. After achieving a steady background noise, 60 µg·mL^−1^ lipopolysaccharide (LPS) was added into the cell mixture to stimulate the release of H_2_O_2_ from cells. Then 500 unit·mL^−1^ catalase was added to decomposing H_2_O_2_. As a control group, LPS and catalase were added to PBS solution without cells. An optimized potential of −0.5 V was applied to the Ag-mSiO_2_ NPs-modified GC electrode for amperometric analysis.

### 2.5. Characterization

High-resolution transmission electron microscopy (HRTEM, CM200UT, Philips, FEI Co., Hillsboro, OR, USA) was used to examine the morphology and distribution of mSiO_2_ NPs and Ag-mSiO_2_ NPs. X-ray diffraction (XRD) pattern was analyzed by an X-ray powder diffractometer (D8 Advance, Bruker, Berlin, Germany). Ultraviolet-visible spectroscopy (UV-vis) absorbance spectra were recorded by UV-vis spectrophotometer (Nanjing Feile Instrument Company, Nanjing, China) with a wavelength range from 300 to 800 nm. 

Cyclic voltammetric and amperometric analysis were performed on a CHI660E electrochemical analyzer (Chenhua Co., Shanghai, China) at room temperature. A bare Glassy Carbon (GC) electrode or Ag-mSiO_2_ NPs modified GC electrode, a Platinum foil electrode and saturated calomel electrode (SCE), were used as the working, the counter and the reference electrode, respectively. Before the detection of H_2_O_2_, the solutions were purged by high purity N_2_ for 30 min to remove oxygen. 

## 3. Results

### 3.1. Characterization of Ag-mSiO_2_ NPs

**Figure 2:**
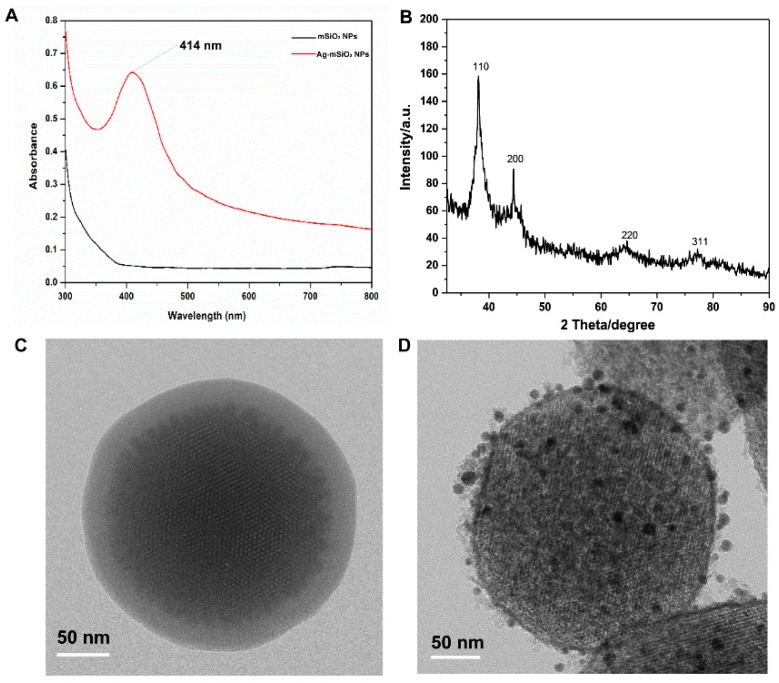
Characterization of mSiO_2_ NPs and Ag-mSiO_2_ NPs. (**A**) UV-vis-NIR absorption spectrum. (**B**) Wide-angle XRD patterns. (**C**) High-resolution transmission electron microscopy (HRTEM) of mSiO_2_ NPs. (**D**) HRTEM of Ag-mSiO_2_ NPs.

Figure 2A shows the UV-vis absorption spectra of mSiO_2_ NPs and Ag-mSiO_2_ NPs. The UV-vis absorbance spectrum of mSiO_2_ NPs at 414 nm was enhanced after Ag NPs decorated. In the wide-angle XRD pattern of the Ag-mSiO_2_ NPs (Figure 2B), four well-resolved diffraction peaks at 2θ values in the range of 30°–90° can be indexed to face-centered cubic (fcc) Ag 111, 200, 220, and 311 reflections [29]. The typical morphology and structure of mSiO_2_ and Ag-mSiO_2_ NPs was observed clearly through HRTEM. Small Ag NPs were doped in the ordered porous framework of spherical Ag-mSiO_2_ NPs (Figure 2C,D), which protect themselves from aggregation. 

### 3.2. Electrocatalytic Reduction of H_2_O_2_

The electrocatalytic activity of the Ag-mSiO_2_ NPs modified GCE toward the H_2_O_2_ reduction was evaluated in PBS (0.2 M, pH 6.8) with a scan rate of 50 mV·s^-1^. Different volumes of Ag-mSiO_2_ NPs (3 μL, 5 μL, 8 μL, and 10 μL) modified on the electrode for better cyclic voltammetric (CV) responses were investigated. The modifier of 8 μL on the surface showed the best response, which was applied for the following experiment (Figure S1). The mechanism of H_2_O_2_ reduced by Ag-doped mesoporous NPs is as follows [24]:

H_2_O_2_ + e^−^ ↔ OH_ads_ + OH^−^
OH_ads_ + OH^−^ ↔ OH^−^
2OH^−^ + 2H^+^ ↔ 2H_2_O

**Figure 3:**
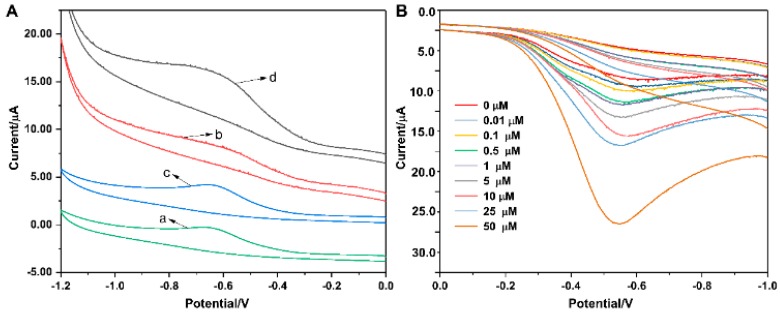
(**A**) Cyclic voltammogram of bare glass carbon electrode (GCE) (a,c) and Ag-mSiO_2_/GCE (b,d) in 0.2 M PBS at pH 6.8 in the absence and the presence of 0.1 mM H_2_O_2_, respectively. (**B**) Current–potential curves of the Ag-mSiO_2_/GCE for electrocatalytic reduction of H_2_O_2_ at the scan rate of 50 mV s^−1^ in 0.2 M PBS solution with different concentrations of H_2_O_2_, respectively.

Figure 2A shows the cyclic voltammogram of bare GC electrode (curve a and c) and Ag-mSiO_2_ modified GC electrode (curve b and d) in PBS (0.2 M, pH 6.8) with the absence or presence of 0.1 mM H_2_O_2_. In the absence of H_2_O_2_, no obvious cathodic current was observed for bare GCE or Ag-mSiO_2_ modified GCE (Figure 3Aa,b). However, after the addition of H_2_O_2_, the reduction current of Ag-mSiO_2_ modified GC electrode (Figure 3Ad) greatly increased, while the response signal of the bare GC electrode was very weak (Figure 3Ac). Cyclic voltammogram of Ag-mSiO_2_ NPs modified GC electrode for H_2_O_2_ reduction at various concentrations of H_2_O_2_ was shown in Figure 3B. The result revealed that the current responses increased with an increased H_2_O_2_ concentration, indicating a good electrocatalytic behavior of Ag-mSiO_2_/GCE towards H_2_O_2_ reduction. 

### 3.3. Amperometric Determination of H_2_O_2_

**Figure 4:**
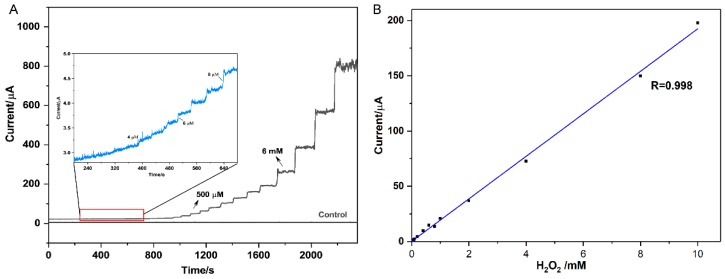
(**A**) Current–time (i−t) data and (**B**) calibration plot for H_2_O_2_ for Ag-mSiO_2_/GC electrode. Inset in (**A**) shows current–time response for 4, 6, and 8 μmoL H_2_O_2_ (arrows in black indicate point of aliquot additions).

The amperometric analysis was used to evaluate the electrocatalytic activity of Ag-mSiO_2_/GC electrode for different concentrations of H_2_O_2_. First of all, the effect of applied potential on amperometric current-time (i−t) curve was studied. Several applied potentials of −0.40, −0.45, −0.50, and −0.55 V was employed to investigate the according amperometric response of Ag-mSiO_2_/GC electrode in PBS (0.2 M, pH 6.8) with the successive addition of 1.0 mM H_2_O_2_. The amperometric response reached a maximum value at a potential of −0.50 V (Figure S2), which was selected for the following amperometric analysis. Figure 4A showed the amperometric i–t curves of Ag-mSiO_2_ NPs/GC electrode with the successive additions of H_2_O_2_ into deoxygenated PBS (0.2 M, pH 6.8) under continuous stirring. Before adding H_2_O_2,_ the amperometric current of Ag-mSiO_2_ NPs/GC electrode was recorded in PBS solution for 200 s to obtain a steady blank control. The Ag-mSiO_2_ NPs/GCE-based sensor could reach a steady-state current within 2 s after a small amount addition of H_2_O_2_, which indicated its fast response for H_2_O_2_ reduction. Additionally, a wide linear range from 4 μM to 10 mM was obtained. The LOD is calculated to be 3 μM (S/N = 3) (Figure 4B). The present H_2_O_2_ biosensor revealed a wide linear range, low LOD, and fast response.

### 3.4. Interferences Study

**Figure 5:**
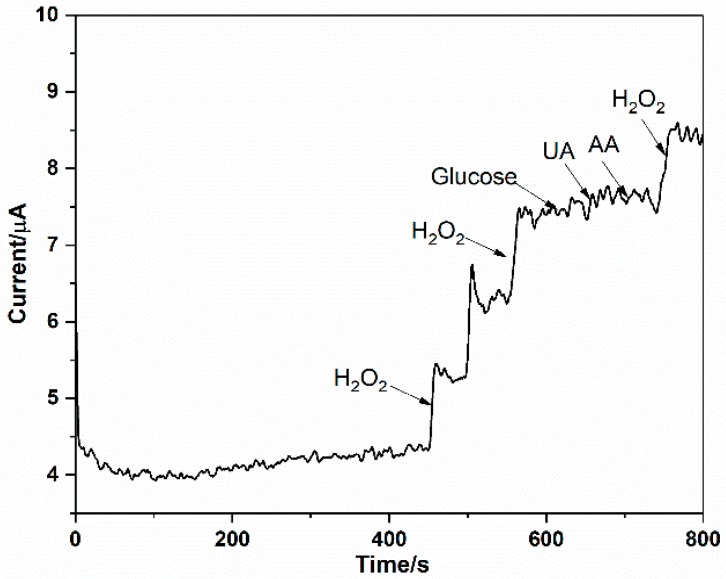
Current-time response curve at Ag-mSiO_2_/GCE for successive injection of H_2_O_2_, glucose, UA, AA in 0.2 M PBS at −0.5 V.

Glucose, ascorbic acid (AA), and uric acid (UA) are three commonly interferents in a physiological system. The anti-interference effect of the Ag-mSiO_2_ NPs /GCE based sensor toward glucose, AA, and UA was investigated. The amperometric responses of the present sensor with sequential addition of 0.05 mM H_2_O_2_, 1 mM glucose, 0.15 mM AA, and 0.5 mM UA are shown in Figure 5. It can be seen that a large amperometric response was achieved with a low concentration of 0.05 mM H_2_O_2_, while negligible current was observed with high concentrations of the other interfering species such as glucose, AA or UA. This showed the present sensor was selective to H_2_O_2_.

### 3.5. Reproducibility and Stability

The reproducibility of Ag-mSiO_2_ NPs modified single electrode was tested for five successive measurements with the relative standard derivation (RSD) in about 2.6%. Moreover, the RSD of current responses of five electrodes was investigated and found to be less than 5%, suggesting the high reproducibility and good precision of our present sensor. Furthermore, the current responses of the sensor decreased to 90.2% of its original value after being stored in the refrigerator at 4 °C for 20 days. The above results indicated the reliability and long-term stability of our fabricated H_2_O_2_ sensor. In addition, we compared the hydrogen peroxided sensing by AgNP impregnated silica NPs in Table 1. H_2_O_2_ sensor reported here has a wide linear range and low detection limit and can detect the H_2_O_2_ released from living cells. 

### 3.6. Determination of H_2_O_2_ Released from Pheochromocytoma Cells

**Figure 6:**
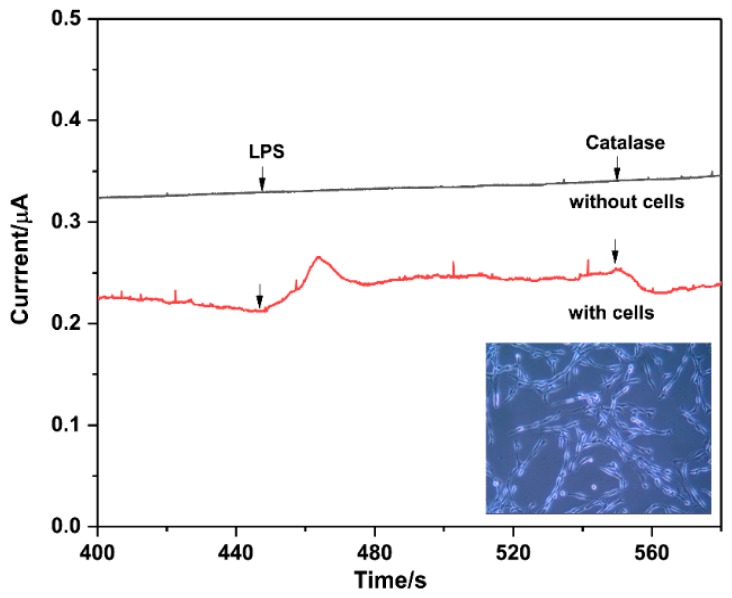
Amperometric responses of the Ag-mSiO_2_/GCE in 0.2 M PBS (pH 6.8) with the addition of LPS and catalase in the absence (upper curve) and presence (lower curve) of PC 12 cells.

Moreover, we performed a study of the real-time determination of H_2_O_2_ released from live cells. For the experiment, about 2 × 10^5^ pheochromocytoma (PC-12) cells in 4 mL PBS (pH = 6.8) with 100 mM glucose were used. LPS (60 μg mL^−1^) was employed to stimulate PC-12 cells to generate H_2_O_2_. After treating with LPS, an obvious current change occurred in solution with cells, while no signal could be observed in that without cells. We also can see from Figure 6 that the current decreased rapidly with an injection of 500 unit·mL^−1^ catalase. As catalase is a selective scavenger of H_2_O_2_, it can be concluded that the increased current in the curve “with cells” was assigned to the reduction of H_2_O_2_. 

## 4. Conclusions

In summary, a highly sensitive and selective enzyme-less sensor of H_2_O_2_ was successfully constructed with Ag-mSiO_2_ NPs/GCE, enabling good electrocatalytic performance toward H_2_O_2_ reduction. The modified electrode exhibited a fast response to H_2_O_2_ concentration variation at an optimized working potential of −0.5 V. A wide linear range from 4 μM to 10 mM with a low LOD of 3 μM was obtained. In addition, the presented sensor showed high selectivity over commonly interfering substances, such as uric acid (UA), ascorbic acid (AA), and glucose. Furthermore, the Ag-mSiO_2_ NPs/GCE based enzyme-less sensor exhibits good reproducibility and long-term stability. Accordingly, the constructed biosensor could be successfully used for the determination of H_2_O_2_ released from live cells. As ROS or H_2_O_2_ has been associated with cell cycle arrest, apoptosis, migration, and inflammation, or the antibacterial activity of certain bacteria such as *Lactobacilli*, our sensor has potential to help understand the role of H_2_O_2_ that plays in the biological and pathological systems. However, for monitoring the ROS or H_2_O_2_ from real samples, the stability of the constructed sensor still needs to be improved with employing more advanced nanomaterials because there is a lot of interferent in real clinical samples. 

## Figures and Tables

**Table 1:** Comparison of the present work with that reported in literature for hydrogen peroxide sensing by Ag NP impregnated silica NPs.

Types of Electrode	Applied Potential (V)	Linear Range (µM)	Detection Limit (µM)	Samples	Reference
AgNP-NH_2_-SBA-15-GCE	−0.4 vs. SCE	0.49–5.3 5.3–124.5	N.A.	PBS buffer	[19]
Ag/SBA-16/CPE	−0.45 vs. Ag|AgCl	20–8000 8000–20,000	2.95	Hair dying cream	[20]
Ag@SiO_2_ YSNs	−0.5 vs. Ag|AgCl	100–15,000	3.5	PBS buffer	[18]
Ag NPs/porous silicon	−0.45 vs. Ag|AgCl	1.65–500	0.45	Hair color oxidant	[10]
Nanoporous silver/Ni wire	−0.35 vs. SCE	10.0–8000	4.80	PBS buffer	[30]
Ag-mSiO_2_ NPs/GCE	−0.5 vs. SCE	4–10,000	3	Live cell	This work

## Supplementary Materials

The following are available online at https://www.mdpi.com/2072-666X/10/4/268/s1, Figure S1: current–potential curves of the Ag-mSiO_2_ NPs/GCE for electrocatalytic reduction of H_2_O_2_ at the scan rate of 50 mV s^−1^ in 0.2 M PBS solution with different modifiers of Ag-mSiO_2_; Figure S2: The amperometric response of Ag-mSiO_2_ nanoparticles/glass carbon electrode (NPs/GCE) (with applied potentials of −0.40, −0.45, −0.50, and −0.55 V) in PBS solution (0.2 M, pH 6.8) upon successive additions of 1.0 mM H_2_O_2_.
