# Supplementary material for: Silver Doped Mesoporous Silica Nanoparticles Based Electrochemical Enzyme-Less Sensor for Determination of H2O2 Released from Live Cells
Source: Micromachines (Basel). 2019 Apr 21;10(4):268. doi: 10.3390/mi10040268 (PMC6523606; doi:10.3390/mi10040268)
Supplement: Supplementary file 1 [file micromachines-10-00268-s001.pdf]

Article

Silver Doped Mesoporous Silica Nanoparticles Based Electrochemical Enzyme-Less Sensor for Determination of H_2O_2 Released from Live Cells

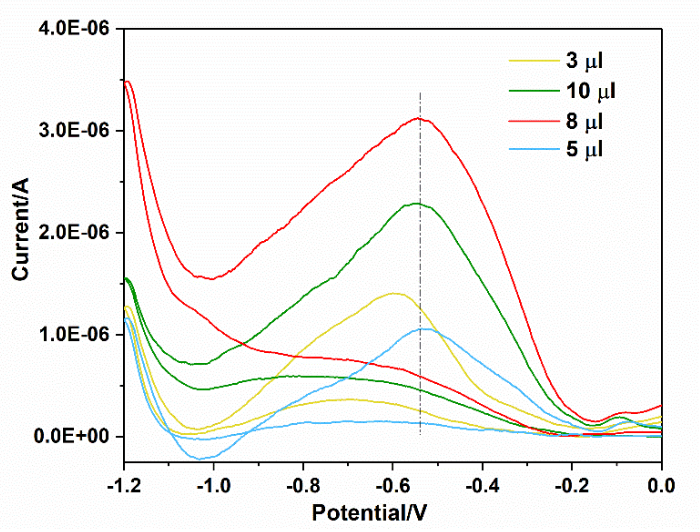

Figure S1. Current–potential curves of the Ag-mSiO_2 nanoparticles/glass carbon electrode (NPs/GCE) for electrocatalytic reduction of H_2O_2 at the scan rate of 50 mV s^{-1} in 0.2 M PBS solution with different modifiers of Ag-mSiO_2 NPs.

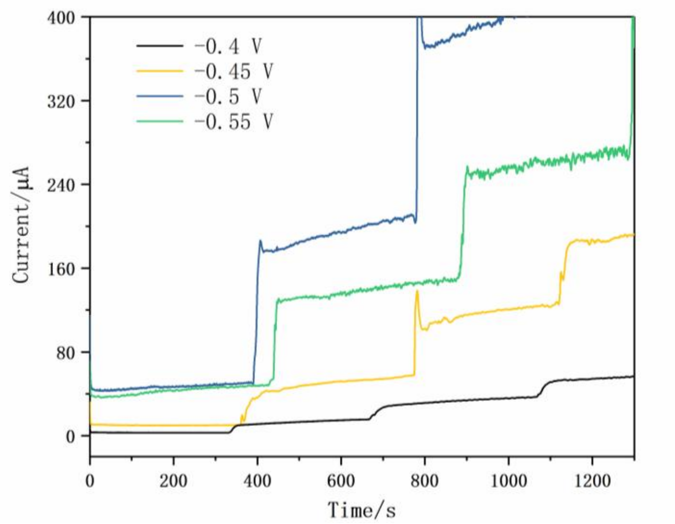

Figure S2. The amperometric response of Ag-mSiO_2 nanoparticles/glass carbon electrode (NPs/GCE) (with applied potentials of −0.40, −0.45, −0.50, and −0.55 V) in PBS solution (0.2 M, pH 6.8) upon successive additions of 1.0 mM H_2O_2.

© 2018 by the author. Licensee MDPI, Basel, Switzerland. This article is an open access article distributed under the terms and conditions of the Creative Commons Attribution (CC BY) license (http://creativecommons.org/licenses/by/4.0/).
